# Impact of the Olig Family on Neurodevelopmental Disorders

**DOI:** 10.3389/fnins.2021.659601

**Published:** 2021-03-30

**Authors:** Jenny Szu, Alexandre Wojcinski, Peng Jiang, Santosh Kesari

**Affiliations:** ^1^Department of Translational Neurosciences and Neurotherapeutics, Saint John’s Cancer Institute, Providence Saint John’s Health Center, Santa Monica, CA, United States; ^2^Department of Cell Biology and Neuroscience, Rutgers University, Piscataway, NJ, United States; ^3^Pacific Neuroscience Institute, Providence Saint John’s Health Center, Santa Monica, CA, United States

**Keywords:** Olig, transcription factor, neurodevelopment, down syndrome, autism spectrum disorder

## Abstract

The *Olig* genes encode members of the basic helix-loop-helix (bHLH) family of transcription factors. Olig1, Olig2, and Olig3 are expressed in both the developing and mature central nervous system (CNS) and strictly regulate cellular specification and differentiation. Extensive studies have established functional roles of *Olig1* and *Olig2* in directing neuronal and glial formation during different stages in development. Recently, Olig2 overexpression was implicated in neurodevelopmental disorders down syndrome (DS) and autism spectrum disorder (ASD) but its influence on cognitive and intellectual defects remains unknown. In this review, we summarize the biological functions of the Olig family and how it uniquely promotes cellular diversity in the CNS. This is followed up with a discussion on how abnormal Olig2 expression impacts brain development and function in DS and ASD. Collectively, the studies described here emphasize vital features of the Olig members and their distinctive potential roles in neurodevelopmental disease states.

## Introduction

The central nervous system (CNS) is composed of various cell types that work synergistically for proper brain function. The complex neural networks of the CNS are strictly governed by neuronal and glial cell development in both space and time. Members of the basic helix-loop-helix (bHLH) transcription factors are critical regulators of neural cell fate specification and differentiation, promoting overall cellular diversity (Dennis et al., 2019). bHLH proteins form heterodimeric complexes that specifically binds to E-box motifs with consensus sequence CANNTG (Bertrand et al., 2002). While neuronal bHLH transcription factors have long been established (Dennis et al., 2019), glial bHLH transcription factors were only recently discovered (Lu et al., 2000; Takebayashi et al., 2000; Zhou et al., 2000). Specifically, oligodendrocyte precursor cells (OPCs), a subtype of glia and precursors to oligodendrocytes (Armada-Moreira et al., 2015), were found to express the novel bHLH transcription factor family of *Olig* genes (Meijer et al., 2012). The Olig bHLH transcription factors consist of three members, Olig1, Olig2, and Olig3, where their expression have been implicated in not only oligodendrocyte differentiation but neuronal and glial cell lineages as well (Lu et al., 2000; Takebayashi et al., 2000; Zhou et al., 2000). While it is evident that these transcription factors have dynamic function during embryonic development, they have also been shown to play critical roles in CNS diseases. In this review, we begin by discussing the known functions of the Olig genes and proteins followed by their influence on neurodevelopmental disorders including down syndrome (DS) and autism spectrum disorder (ASD). We then conclude with a section on potential therapeutic strategies targeting the Olig proteins to rescue DS and ASD phenotype. Since its discovery, the functions of the Olig family and characterization of the Olig genes and the proteins they encode have been performed in various species. Our review will primarily focus on Olig transcription factors in studies using rodent models and human pluripotent stem cell (hPSC) models.

### Expression Patterns of *Olig* Genes in the Developing CNS

In early 2000, three independent groups identified the Olig family of transcription factors (Lu et al., 2000; Takebayashi et al., 2000; Zhou et al., 2000). Olig1 and Olig2 bHLH transcription factors were first identified from human genomic sequences within a single bacterial artificial chromosome (BAC) clone derived from chromosome 21q22 (syntenic to mouse chromosome 16) (Takebayashi et al., 2000; Zhou et al., 2000). Olig3 was found in a BAC clone obtained from human chromosome 6q24 (syntenic to mouse chromosome 10) (Takebayashi et al., 2000). The deduced amino acid sequences are nearly identical in the bHLH domain across the Olig family (Lu et al., 2000; Zhou et al., 2000; Schebesta and Serluca, 2009) and the bHLH domain of Olig1 and Olig2 were found to be structurally similar to neuronal transcription factors NeuroD, NeuroD-related factor (Ndrf), and Neurogenin-2 (Ngn2) (Lu et al., 2000; Takebayashi et al., 2000). The loop region, however, was found to be significantly divergent (Lu et al., 2000; Takebayashi et al., 2000; Zhou et al., 2000). At the protein level, Olig2 was found to be more similar to Olig3 (Meijer et al., 2012). While all three *Olig* genes are expressed in the developing CNS (Lu et al., 2000; Takebayashi et al., 2000; Zhou et al., 2000), *Olig1* and *Olig2* are widely expressed in the adult brain with *Olig3* weakly expressed in skeletal muscle, testis, and submaxillary gland (Takebayashi et al., 2000).

Expression of *Olig* genes in the developing spinal cord has been well characterized in rodent and chick embryos. *In situ* hybridization revealed expression of *Olig1* and *Olig2* in the ventral spinal cord as early as embryonic day (E) 9.5 with *Olig2* displaying stronger expression (Lu et al., 2000; Zhou et al., 2000). Interestingly, the expression of *Olig1/2* appear prior to the earliest OPC markers *platelet-derived growth factor alpha* (*PDGFR*α), *cyclic nucleotide phosphodiesterase* (*CNP*), and *proteolipid protein* (*DM20/PLP*) (Lu et al., 2000; Takebayashi et al., 2000; Zhou et al., 2000) which are first detected at E12.5 (Lu et al., 2000). As development progresses, *Olig2* expression becomes restricted to the progenitors of motor neurons (pMN) domain, a narrow band within the ventral neuroepithelium of the spinal cord, and expression is further increased by E12.0. Similarly, *Olig1* expression is also confined to the pMN domain by E10.5 (Zhou et al., 2000). By E14.5, *Olig1/2*^+^ cells were found scattered within the ventral mantle zone, indicative of OPCs migrating from their site of origin (Lu et al., 2000; Takebayashi et al., 2000; Zhou et al., 2000; Tekki-Kessaris et al., 2001).

*Olig* genes have also been detected in the embryonic and postnatal brain. In the mouse embryonic brain, *Olig1/2* expression was primarily found in the midbrain and hindbrain at E10.5–E14.5 (Takebayashi et al., 2000; Zhou et al., 2000). *Olig2*^+^ cells were particularly abundant in the ventricular zone (VZ) and subventricular zone (SVZ) of the lateral (LGE) and medial (MGE) ganglionic eminences (Takebayashi et al., 2000; Tekki-Kessaris et al., 2001) with few cells expressing *Olig1* (Takebayashi et al., 2000). The VZ and SVZ are known sites of neurogenesis (Götz and Huttner, 2005), thus this hints at a possible role of *Olig2* in neuronal differentiation and fate specification. In the postnatal brain, *Olig1/2*^+^ cells with oligodendrocyte morphology were observed in the corpus callosum, hippocampus, and cerebellar white matter (Lu et al., 2000; Takebayashi et al., 2000). *Olig1/2* expressing cells were also found scattered throughout the gray matter suggesting OPC migration (Lu et al., 2000; Takebayashi et al., 2000; Zhou et al., 2000). The optic nerve was also abundant with cells expressing *Olig1/2* (Lu et al., 2000; Takebayashi et al., 2000; Zhou et al., 2000). *In situ* hybridization experiments in dissociated primary cultures of P5 optic nerve showed that *Olig1* and *Olig2* were expressed by bipolar cells (Zhou et al., 2000).

While *Olig1* and *Olig2* expression are well characterized, little is known about Olig3. Early *in situ* hybridization experiments with mouse embryos revealed *Olig3* expression in the dorsal neural tube at E9.25 and E10.5. *Olig3* is transiently expressed throughout embryonic development and limited to the CNS. From E10.5 to E12.5, *Olig3*^+^ cells were found located in the dorsal spinal cord with ventral expression clusters observed at E11.5. *Olig3* was also found to be expressed in the hindbrain (Takebayashi et al., 2002b; Storm et al., 2009; Lowenstein et al., 2021) and the ventricular zone of the dorsal thalamus of the forebrain (Takebayashi et al., 2002b). Expression of *Olig3* during cerebellar development was recently characterized (Lowenstein et al., 2021). In the dorsal part of rhombomere 1 contains two germinal zones, the rhombic lip and ventricular zone, which produces glutamatergic and GABAergic neurons, respectively (Yamada et al., 2014). In the rhombic lip, Olig3^+^ cells first appear as early as E10.5 and peaks by E11.5 and is noticeably absent by E14.5. Coexpression of Olig3^+^ with Sox2 was apparent in over 98% of the cells between E10.5 and E12.5 and about a third of Olig3^+^ cells coexpress with atonal homolog one transcription factor (Atoh1) (Lowenstein et al., 2021), a transcription factor required for generation of glutamatergic neurons (Yamada et al., 2014). In the ventricular zone, Olig3-expressing cells first emerge at E11.5, peaks by E12.5, and declines by E14.5. About 59% of Olig3^+^ cells also coexpress with Sox2 between E11.5 and E12.5. Roughly 52% of Olig3^+^ cells coexpress the pancreas-specific transcription factor 1a (Ptf1a) (Lowenstein et al., 2021), which regulates production of GABAergic neurons (Yamada et al., 2014) while 41% coexpress with Foxp2, a marker for postmitotic Purkinje cells (Lowenstein et al., 2021).

### Hedgehog Signaling Regulates *Olig* Expression During Development

Several studies have elegantly demonstrated that expression of *Olig1* and *Olig2* in mice and humans are regulated by the ventralizing signal Sonic hedgehog (Shh) (Lu et al., 2000; Tekki-Kessaris et al., 2001; Billon et al., 2002; Hu et al., 2009), which is produced by both the floorplate and the notochord (Jeong and Epstein, 2003). *In vivo* regulation of *Olig1* by Shh was determined using transgenic mice ectopically expressing *Shh* in the dorsal midline (*Shh-Tg*). *Shh-Tg* mice exhibited induction of both *Olig1* and *Olig2* as well as *PDGFR*α in regions next to the ectopic ventricular zone at E14.5. Cells expressing O4, a marker for OPCs, were also observed in similar areas at E17.5 indicative of *Olig1* regulation by Shh. Treatment of primary neuroepithelial cell cultures from E14.5 rat cortex with recombinant Shh also led to ∼10-fold rapid upregulation of *Olig1*. Finally, *in situ* hybridization at E10.5 revealed Shh expression in the ventral diencephalon, telencephalon, and the zona limitans intrathalamica (zli). In wildtype (WT) embryos, strong expression of *Olig1 and Olig2* were observed in the zli as compared to *Shh* mutant embryos where *Olig1* and *Olig2* expression were largely absent in CNS. These findings suggest that *Olig1* and *Olig2* can act as Shh transcriptional targets in the forebrain (Lu et al., 2000).

Shh signaling was also shown to be required for the induction and maintenance of Olig2 in human embryonic stem cells (hESCs). First, expression of Olig2 was shown to be induced in a Shh-dependent manner where hESC cultures treated with purmorphamine, an activator of hedgehog signaling pathway, resulted in significantly earlier and increased expression of Olig^+^ progenitors. Furthermore, induction of human pre-OPCs, progenitors that express OPC marker Nkx2.2 but lack PDGFRα, NG2, and Sox10, were also found to be dependent on Shh signaling. hESCs treated with purmorphamine led to generation of Olig2^+^Nkx2.2^+^ pre-OPCs, however, cells lacking Olig2 and/or Nkx2.2 expression was noted when hESCs were treated with cyclopamine, an endogenous Shh blocker. Interestingly, treatment with or without purmorphamine resulted in cells that were positive for Olig2 and Nkx2.2, suggesting that exogenous Shh signaling is not required to maintain co-expression of Olig2 and Nkx2.2 in pre-OPCs and to further promote OPC generation (Hu et al., 2009). Altogether, these findings indicate that Shh signaling is required to maintain coexpression of Olig2 and Nkx2.2 in pre-OPCs and that *Olig2* is a downstream target of Shh.

Regulation of *Olig1* and *Olig2* by Shh was also confirmed using genetically engineered mouse embryonic stem cells (mESCs). mESCs maintained with fibroblast growth factor-2 (FGF-2) resulted in less than 12% of the cells expressing *Olig1* and *Olig2*. However, the addition of Shh with FGF-2 in culture for 5 days resulted in 20–45 and 23% of OPCs expressing *Olig1* and *Olig2*, respectively. Furthermore, mESCs cultured for 8 days in the same condition increased *Olig2* expressing OPCs to 40–85% (Billon et al., 2002). In a separate study, induction of Olig2^+^ cells by Shh-mediated FGF-2 signaling was observed in CNS stem cells *in vitro*. E14.5 neural tube cells plated for 3 days with Shh agonist either in the presence or absence of FGF induced Olig2 expression without promoting proliferation. When a small-molecule Shh antagonist was applied, a 3.6-fold reduction in Olig2^+^ cells were observed. These studies suggest that induction of Olig2 spinal cord progenitors requires FGF signaling that is controlled in part by Shh-dependent mechanism (Gabay et al., 2003).

## Cell Fate Specficiation

**FIGURE 1:**
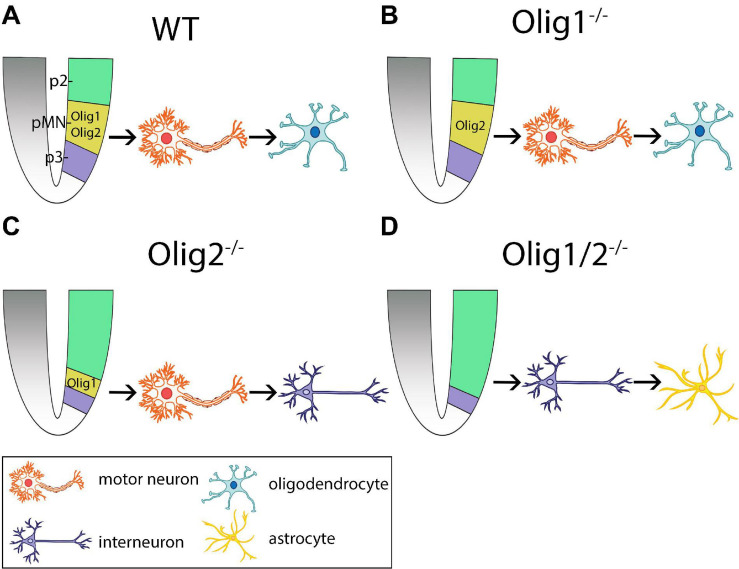
Schematic of cellular patterning in the mammalian spinal cord after loss of *Olig* function. **(A)** *Olig1* and *Olig2* expression is restricted to the progenitors of motor neurons (pMN) domain in wildtype (WT) mice. Here, motor neurons are generated first followed by generation of oligodendrocytes. **(B)** Loss of *Olig1* results in similar cellular differentiation and specification as WT mice. **(C)** Loss of *Olig2* results in partial expansion of the p2 domain into the pMN domain. Here, motor neurons are generated prior to the generation of interneurons. **(D)** Loss of *Olig1/2* results in complete invasion of the pMN domain by the p2 domain. Here, interneurons are generated prior to the generation of astrocytes. pMN = progenitors of motor neurons.

**FIGURE 2:**
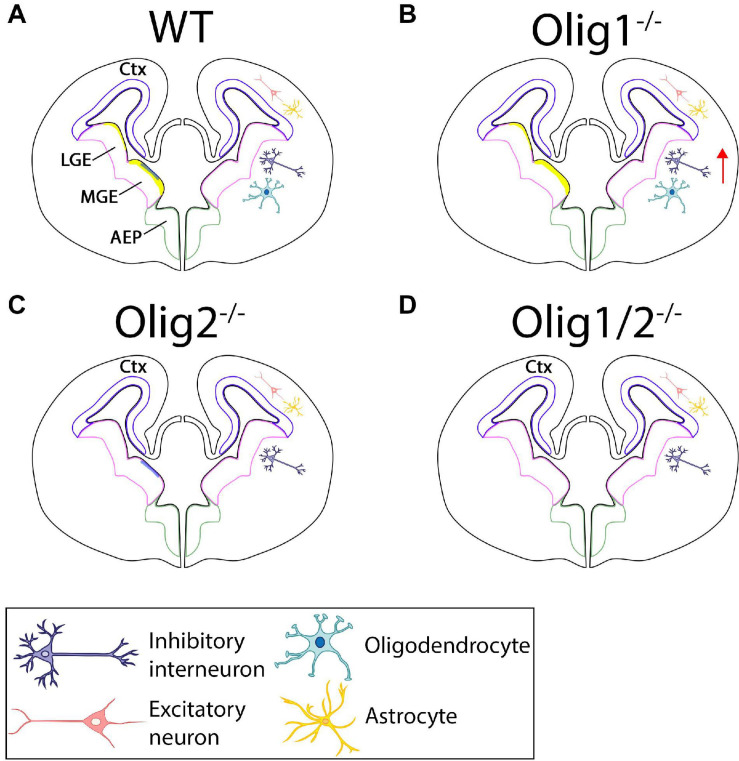
Schematic of cellular patterning in the mammalian forebrain after loss of *Olig* function. **(A)** In the WT forebrain, *Olig1* and *Olig2* are expressed in the LGE and MGE. *Olig2* (yellow) is highly upregulated along the borders of the MGE with little expression in the LGE. *Olig1* (blue) is slightly expressed in the MGE. Astrocytes and excitatory neurons are generated in the ctx while interneurons and oligodendrocytes are formed from the LGE and MGE. **(B)** Loss of *Olig1* results in an increase in interneurons. **(C)** Loss of *Olig2* results in loss of oligodendrocytes. **(D)** Loss of *Olig1* and *Olig2* results in only loss of oligodendrocytes. Ctx = cortex, LGE = lateral ganglionic eminences, MGE = medial ganglionic eminences.

The molecular underpinnings that regulate cell fate is governed in part by the novel Olig family of bHLH transcription factors encoded by its related *Olig* genes. The role of *Olig* genes in cell fate specification and differentiation is a multifaceted one. Together with other transcription factors, cellular identity at topologically defined regions is beautifully coordinated by *Olig1* and *Olig2* to allow construction of dynamic neural circuits. Below, we summarize how the Olig family participates in the development of overall cellular diversity in the CNS (Figures 1, 2).

### Oligodendrocyte Fate Specification

As its name suggests, the *Olig* genes are responsible for the development of oligodendrocytes or oligodendrogenesis. In the developing CNS, the *Olig* genes, specifically *Olig1* and *Olig2*, are explicitly expressed in OPCs in a highly restrictive area in the VZ which later migrate to the gray matter (Lu et al., 2000; Takebayashi et al., 2000; Zhou et al., 2000; Anderson et al., 2002). Studies have also shown that *Olig1* primarily contributes to oligodendrocyte formation and maturation in the brain, whereas *Olig2* is mainly required for oligodendrocyte development in the spinal cord (Lu et al., 2002; Ross et al., 2003; Meijer et al., 2012).

#### Oligodendrocyte Differentiation in the Brain

Oligodendrocyte generation in the brain, compared to the spinal cord, is not well understood. The commitment of *Olig1* to initiate oligodendrocyte differentiation in the brain was established in various gain-of-function experiments. Ectopic expression of *Olig1* in primary cultures of rat cortical progenitor cells was shown to stimulate the early oligodendrocyte marker, NG2. An ∼3-fold increase in NG2 was observed in E13.5 rat cortical progenitor cells infected with *Olig1*-carrying adenovirus compared with cells infected with the control virus (Lu et al., 2000). The upregulation of NG2^+^ cells may be attributed to Shh signaling. In fact, an abundance of NG2^+^ cells were observed in E10.5 mouse ventral forebrain and E15.5 rat neocortex 6–8 days in culture. The number of NG2-expressing cells was however drastically reduced in the presence of Shh inhibitor cyclopamine (Tekki-Kessaris et al., 2001). Promotion of oligodendrocyte development by *Olig1* was also shown *in vivo*. As expected, *PDGFR*α and *Sox10* expression are unaltered in the postnatal brains of *Olig1* heterozygotes and *Olig1*-null mice (Xin et al., 2005; de Faria et al., 2014) as Olig1 protein is detected after oligodendrocyte lineage specification (Gokhan et al., 2005; Fu et al., 2009). Additionally, ectopic expression of *Olig1* induced by retroviral infection resulted in a dramatic population of oligodendrocytes in both the gray and white matter at P21, whereas control retrovirus largely developed into neurons and astrocytes (Lu et al., 2001).

Because myelin production is a key function of oligodendrocytes the regulation of myelin may aid in elucidating the role of Olig1 in the development and maturation of oligodendrocytes in the brain. Behaviorally, *Olig1*-null mice displayed characteristics reminiscent of aberrant myelin sheaths such as abnormal limb clasping, tremors, ataxia, and seizures (Xin et al., 2005). Not surprisingly, expression of *myelin basic protein* (*MBP*), *DM20/PLP*, and *CNP*, major myelin genes and markers for mature oligodendrocytes, were absent in these mice compared to WT and *Olig1* heterozygotes (Xin et al., 2005; de Faria et al., 2014). Interestingly, OPCs in *Olig1* null mutants can successfully differentiate and make contact with axons, however, fail to form any myelin (Xin et al., 2005). These findings are largely attributed to deficits in Olig1 transcription factor in myelinating cells. During development, Olig1 is transported from the nucleus to the cytoplasm where cells progressively mature into MBP^+^ oligodendrocytes. The relocalization of Olig1 was found to be essential for remyelination. In fact, nuclear Olig1 was detected in demyelinated mouse brains and in postmortem brain tissue from patients with multiple sclerosis (MS). Furthermore, induction of MBP is delayed after cuprizone-induced demyelination in mice (Arnett et al., 2004). These findings indicate that Olig1 is not only a critical regulator of oligodendrocyte maturation but confirm its role in myelinogenesis.

The function of oligodendrocyte differentiation in the brain by *Olig2* is strikingly different compared to *Olig1*. Compared to WT mice, the ventral forebrain of *Olig2* null mice displayed a near complete loss of the *PDGFR*α- and *Sox10*-expressing OPCs. On the other hand, expression of *PDGFR*α and *Sox10* was largely unaffected in the hindbrain/midbrain of *Olig2* null mice compared to WT mice. Remarkably, an upregulation of *Olig1* was observed in the hindbrains of *Olig2* null mice compared to WT mice suggesting a possible compensatory mechanism (Lu et al., 2002; Takebayashi et al., 2002a). Indeed, *Olig1/2* double mutants displayed neither OPCs nor mature oligodendrocytes in the hindbrains between E13.0 and E.18.5. Compared to WT or heterozygous mice, *Olig1/2* double null mice displayed complete loss of *PDGFR*α, *Sox10*, and *MBP* in all regions of the brain (Zhou and Anderson, 2002). Together, these studies suggest *Olig2* functions in oligodendrocyte differentiation in a region-specific manner.

#### Oligodendrocyte Specification in the Spinal Cord

In contrast to the brain, *Olig1* plays a smaller role in oligodendrocyte development in the spinal cord. In the developing spinal cord, mice lacking *Olig1* showed delayed oligodendrocyte differentiation and reduced expression of *Olig2*, *Sox10*, *MBP*, and *DM20/PLP* (Lu et al., 2002; Xin et al., 2005; de Faria et al., 2014). Interestingly, however, the timing of *PDGFR*α expression was unaffected (Lu et al., 2002). Low levels of *MBP* and *PLP* expression was detected at E18.5 confirming that *Olig1* is not a critical component of oligodendrocyte differentiation in the developing spinal cord. By P4, the numbers of *MBP*^+^ and *PLP*^+^ cells in *Olig1* mutant mice were comparable to those of control mice (de Faria et al., 2014). Moreover, *Olig1* null mice was capable of developing normal oligodendrocytes by P30 (Lu et al., 2002) further supporting the role of *Olig1* in the maturation of oligodendrocytes.

As mentioned previously, *Olig1* was shown to be regulated by Shh signaling in mice ectopically expressing Shh in the dorsal midline. *Shh-Tg* mice did not express *DM20/PLP* at E14.5, however, O4^+^ cells were observed adjacent to the ectopic ventricular zone which may be attributed to *Olig2*. It has been suggested that *Olig2* can partially compensate for the lack of *Olig1*, and vice versa (Zhou and Anderson, 2002), however, evidence for this compensatory mechanism is lacking. More importantly, no significant differences in *Olig2* expression were detected in the spinal cord between *Olig1* null mice, *Olig1* heterozygotes, and WT controls (de Faria et al., 2014). Rather, *Olig2* was downregulated in the white matter tracts of *Olig1* null mice compared to *Olig1* heterozygotes (Xin et al., 2005). These findings refute the idea that *Olig2* can compensate for the loss of *Olig1* in the spinal cord. Additionally, while the functional role of *Olig3* has yet to be determined, the possibility of its contribution to oligodendrogenesis cannot be ruled out.

*Olig2* plays a pivotal and more dominant role in oligodendrocyte development in the spinal cord. Early studies found severe ablation of oligodendrocyte formation in *Olig2* null mice and complete loss of O4-expressing cells in mouse caudal spinal cord explants cultured to the equivalent of P6 (Lu et al., 2002). Failure of oligodendrocyte development as evidenced by lack of PDGFRα^+^ and NG2^+^ oligodendrocyte progenitors was also observed in *Olig2*^–/–^ spinal cord at E14.5 (Takebayashi et al., 2002a). *Olig1/2* double mutants also lacked expression of early oligodendrocyte markers *PDGFR*α and *Sox10* and mature oligodendrocyte markers *MBP* and *DM20/PLP* (Lu et al., 2002; Zhou and Anderson, 2002). On the other hand, compared to WT mice, heterozygotes did not display a decrease in *PDGFR*α^+^ OPCs at E16.5 and expression of *MBP* and *DM20/PLP* was normal at E16.5 and P8. Interestingly, a significant reduction in *MBP* and *DM20/PLP* was observed between E18.5 and P0 in heterozygotes compared to WT mice suggesting that a full dosage of *Olig* genes is required for the progression of oligodendrocyte differentiation (Zhou and Anderson, 2002).

Oligodendrocyte differentiation is not solely dependent on Olig2. Several studies have shown that oligodendrocyte specification is promoted by the cooperation of both Olig2 and the homeobox protein Nkx2.2 (Zhou et al., 2001; Fu et al., 2002; Liu et al., 2007). Early in development, *Olig2* and *Nkx2.2* are restricted to their respective ventricular zone domains. Specifically, *Olig2* resides in the pMN domain and *Nkx2.2* inhabits the p3 domain (Holz et al., 2010). Before oligodendrogenesis, overlapping of the two domains are temporally distinct between species. In the chick, *Nkx2.2*^+^ cells migrate into the pMN domain between E5.0 and E7.0 prior to the production of *Olig2^+^PDGFRα^+^* OPCs (Zhou et al., 2001; Fu et al., 2002). In the mouse however, merging of the p3 and pMN domain occurs ∼2 days after *Olig2*^+^ OPCs have migrated into surrounding regions, thus many of these cells do not coexpress *Nkx2.2* during the early stages of oligodendrogenesis (Fu et al., 2002). The integration of the *Nkx2.2* p3 domain with the dorsal *Olig2* pMN domain is attributed to the neuron-glial fate switch where *Nkx2.2* OPCs may gain *Olig2* expression and adopt a new function to develop oligodendrocytes after neurogenesis ceases (Kessaris et al., 2001; Zhou et al., 2001; Fu et al., 2002). To confirm this, spinal cords from E8.5 chicks (Zhou et al., 2001) and E13.5 mice (Fu et al., 2002) were dissociated and cultured. In dissociated chick spinal cords, 98% of *Olig2*^+^*Nkx2.2*^+^ cells also coexpressed O4. By E10.0, these cells were found to express the mature oligodendrocyte marker myelin-associated glycoprotein (MAG) in the white matter (Zhou et al., 2001). In dissociated mice spinal cords 2 days in culture, about 45% of cells were found to express both *Olig2* and *Nkx2.2* although *Nkx2.2* expression was generally weak. After 5 days *in vitro*, 85% of *Olig2*^+^ cells exhibited strong *Nkx2.2* expression (Fu et al., 2002). These findings imply that *Olig2*^+^ OPCs in chick and mice acquire *Nkx2.2* expression albeit at different times.

Additional evidence that *Nkx2.2* and *Olig2* synergistically regulate oligodendrocyte production can be observed through misexpression studies. E2.0 chick spinal cord were electroporated *in ovo* with replication-competent RCASBP(B) plasmids containing *Olig2* or *Nkx2*.2 genes either singly or in combination. The embryos were then harvested at E6 when OPCs begin to appear in the spinal cord. Coelectroporation of *Olig2* and *Nkx2.2* resulted in a dramatic induction of Sox10, PDGFRα, MBP, and PLP/DM20 indicative of ectopic and precocious oligodendrocyte differentiation. None of these oligodendrocyte markers were expressed when *Olig2* or *Nkx2.2* were electroporated alone in the dorsal spinal cord. When *Olig2* was electroporated in the ventral spinal cord early oligodendrocyte differentiation was observed in the overlapping domain of endogenous *Nkx2.2*. Interestingly, electroporation of *Nkx2.2* in the pMN domain strongly repressed endogenous *Olig2* which was rescued with exogenous *Olig2* (Zhou et al., 2001). These findings indicate that the combination of Olig2 and Nkx2.2 transcription factors have a functional role in activating oligodendrocyte differentiation.

### Neurogenesis

During development, specific neuronal subtypes emerge from progenitor cells along the dorsal-ventral axis of the neural tube and patterning of specific neuronal subtypes is governed by various transcription factors (Briscoe and Ericson, 2001). While both *Olig1* and *Olig2* contributes to oligodendrogenesis, only *Olig2* is implicated in the generation of neurons. Evidence of this comes from both the spatial and temporal expression of *Olig2*. In the developing spinal cord, *Olig2* is distinctly expressed in the pMN domain (Takebayashi et al., 2000; Zhou et al., 2000; Lu et al., 2002), a site for motor neuron generation (Takebayashi et al., 2000; Novitch et al., 2001). Whereas in the developing brain, Olig2^+^ progenitor cells were observed in the hindbrain where motor neurons are formed (Mizuguchi et al., 2001; Zannino and Appel, 2009; Zannino et al., 2012) as well as in the VZ and SVZ of the LGE and MGE (Takebayashi et al., 2000) where interneurons are generated (Casarosa et al., 1999; Lavdas et al., 1999; Stenman et al., 2003). The earliest *Olig2* expression also precedes the generation of motor neurons and interneurons (Lu et al., 2000; Takebayashi et al., 2000). For instance, *Olig2* expression was observed prior to the expression of *Mash1*, a neuronal lineage marker (Takebayashi et al., 2000). Thus, the expression of *Olig2* overlaps the timeline of neurogenesis strongly supports the role of *Olig2* in the neuronal specification. Distinct cerebellar neuronal subtypes arising from Olig3^+^ progenitors have also been characterized. Long-term lineage tracing studies revealed that Olig3^+^ progenitors generate the earliest set of Purkinje cells in the ventricular zone and deep cerebellar nuclei (DCN) neurons in the rhombic lip and external granule layer cells at a later time.

#### Motor Neuron Differentiation

Initial studies demonstrated that motor neuron differentiation from ventral progenitor cells in the pMN domain is controlled by its interaction with the neural bHLH transcription factor Ngn2. In the developing spinal cord, Olig2 and Ngn2 were shown to be expressed in a region-specific manner. Specifically, restriction of Olig2 expression in the pMN is controlled by expression of a group homeodomain (HD) proteins in adjacent domains of the neural tube (Mizuguchi et al., 2001; Novitch et al., 2001) that participates in generation of interneurons (Briscoe et al., 2000; Jessell, 2000). At E10.5, Olig2^+^ and Nng2^+^ cells were detected in the ventral domains of the neural tube with overlapping expression observed by E11.5. Coexpression of Olig2 and Ngn2 were observed in many cells by E12.5, a stage where neuronal subtypes are actively generated (Mizuguchi et al., 2001). At E12.5, Olig2^+^ progenitors were closely associated to HB9 and Isl1 (Mizuguchi et al., 2001), defined markers for motor neurons (Pfaff et al., 1996; Arber et al., 1999). At this time, scattered Nng2^+^ cells were observed in the dorsal neural tube, however most remained in the ventral region. Additionally, expression of Olig1 was not detected from E10.5 to E12.5 when motor neurons are generated. However, Olig1 expression was observed at E14.5 when motor neuron generation is near completion and oligodendrocyte formation begins (Mizuguchi et al., 2001). Together, these results not only support the dual role of Olig2 and Ngn2 in neurogenesis but also confirm the sequential transformation of neurons to glial cells (which is regulated by the presence of Olig1).

A correlation between Olig2 and Ngn2 in motor neuron generation was also observed in the hindbrain. At E12.5, bilateral coexpression of Olig2 and Ngn2 was detected in the ventral domain. In the hindbrain, two specific types of motor neurons are generated: (1) motor neurons that extends their axons ventrally (vMNs) and express HB9 and (2) motor neurons that projects their axons dorsally (dMNs) and express Phox2b. Both VMNs and dMNs express Isl1. Olig2 and Ngn2-expressing progenitors are observed medial to HB9^+^ vMNs but are isolated from Phox2b^+^ dMNs indicating that Olig2^+^ and Ngn2^+^ cells are associated with vMNs. To establish that production of vMNs is regulated by Olig2 and Ngn2, Pax6 mutant rats (*Sey/Sey*) were used (Mizuguchi et al., 2001). Pax6 regulates vMN specification (Osumi et al., 1997; Takahashi and Osumi, 2002) and *Sey/Sey* embryos were shown to express ectopic dMNs in the hindbrain (Ericson et al., 1997; Osumi et al., 1997). In Pax6 mutant rats, loss of Olig2, Ngn2, and HB9 expression were observed in the vMNs. Instead, Phobx2b and Mash1 expression was induced in this progenitor domain. These findings demonstrate that Olig2 and Ngn2, together, is involved in vMN fate and that Olig2 and Ngn2 coexpression in the hindbrain is regulated by Pax6 (Mizuguchi et al., 2001).

The role of Olig2 and Ngn2 in motor neuron fate was further evidenced by gain-of-function studies. Misexpression of Ngn2 induced ectopic cells expressing pan-neuronal marker β-tubulin and the postmitotic neuron-specific neurofilament-associated antigen but did not induce expression of motor neuron markers (Mizuguchi et al., 2001). Unlike Ngn2, Olig2 overexpression did not induce ectopic neurogenesis but did stimulate ectopic cells to express various motor neuron markers including MNR2, Lim3, Isl1, and Isl2 in a region dorsal to the motor neuron domain (Mizuguchi et al., 2001; Novitch et al., 2001). Specifically, a 1.7-fold increase in ectopic MNR2/HB9^+^ motor neurons was detected in the ventral neural tube at Hamburger-Hamilton (HH) stages 14–15. Dorsal expansion of ectopic Ngn2^+^ cells was also detected when Olig2 was misexpressed (Novitch et al., 2001). Furthermore, overexpression of Olig2 reduced Pax7 and Irx3 while ectopic expression of Ngn2 suppressed Pax7 and Irx3 (Mizuguchi et al., 2001). Pax7 is involved in restricting neuronal identity in the spinal cord (Mansouri and Gruss, 1998) and Irx3 is known to inhibit motor neuron differentiation (Briscoe et al., 2000). The dysregulation of Pax7 and Irx3 expression may be a result of increased expression of MNR2 contributed by Olig2 and Ngn2 to induce ectopic motor neurons in the dorsal neural tube. Interestingly, the combinatorial misexpression of both Olig2 and Ngn2 resulted in many cells expressing a variety of motor neuron markers, including MNR2/HB9, Lim3, Isl1, and SC1, in the extreme dorsal region of the neural tube. Moreover, 4.1-fold increase in MNR2/HB9^+^ cells were found scattered in both the dorsal and ventral regions. During active motor neuron generation (HH stages 19–20), misexpression of Olig2 alone and of both Olig2 and Ngn2 led to a 1.5-fold and 2.1-fold increase in MNR2/HB9^+^ cells, respectively (Mizuguchi et al., 2001). Altogether, these findings suggest that Olig2 with Ngn2 together regulates motor neuron generation by promoting motor neuron subtype identities such as MNR2, HB9, and Lim3.

Loss-of-function studies have also validated the function of Olig2 in motor neuron specification. *Olig2*^±^ mice displayed normal morphology and were viable while *Olig2*^–/–^ pups died during birth and preserved their *in utero* posture. Further examination of *Olig2*^–/–^ pups found no motor neurons in the ventral horn at E18.5. Additionally, few Isl1^+^/HB9^+^ motor neurons were detected in the spinal cord of *Olig2*^–/–^ embryos at E10.5 indicating that motor neurons were being generated but unable to survive (Takebayashi et al., 2002a). Similarly, in the hindbrain, *Olig2* null mice were completely devoid of Is1^+^/Hb9^+^ somatic motor neurons (Lu et al., 2002). Additionally, *Olig2*^–/–^ mice exhibited an expansion and increase in Chx10^+^ V2 interneurons in the ventral spinal cord at E10.5 and E12.5 whereas ectopic expression of Olig2 inhibited Chx10 expressing V2 interneurons (Lu et al., 2002). In a separate study, E10.5 *Olig1/2* double mutant mice displayed abolishment of Isl1/2^+^ and Hb9^+^ spinal cord motor neurons compared to heterozygous and WT mice which retained the same number of these motor neurons. At E13.5, neither somatic nor visceral motor neurons were detected in the spinal cord of these mice. Interestingly, the absence of both *Olig1* and *Olig2* resulted in ∼80% increase in Chx10^+^ V2 interneurons that expanded into the domain primarily occupied by motor neurons, whereas the number and distribution of En1^+^ V1 interneurons and Ngn3^+^ V3 interneurons are relatively unchanged. To confirm that *Olig1/2* progenitor cells in the pMN give rise to motor neurons, *Olig2* knockin marker *histone*-GFP (hGFP) was used as a short-term lineage tracer. In *Olig1*^±^*Olig2*^±^ heterozygotes, Olig2-hGFP^+^ precursors in the pMN gave rise to Isl1/2^+^ motor neurons but generated Chx10^+^ V2 interneurons in *Olig1*^–/–^*Olig2*^–/–^ homozygotes (Zhou and Anderson, 2002). These findings establish that Olig2 is responsible for motor neuron cell fate acquisition.

Studies have also demonstrated that Olig2 may act as a transcriptional repressor. Using a Gal4-repression/activation assay, the level of Olig2 activity was measured in COS-1 cells cotransfected with either full-length Olig2 protein fused to yeast Gal4 DNA binding domain (Gal4-Olig2) or with transcriptional activator MyoD (Gal4-MyoD). Transcription was reduced by 8-fold and 6-fold in Gal4-Olig2 and Gal4-MyoD, respectively. When Olig2 bHLH domain was fused to the repressor domain of the Drosophila Engrailed protein (Olig2-EnR) neural tube patterning mirrored that of full-length Olig2 and *Irx3* expression was repressed. Ectopic MNR2, Lim3, or HB9 expression was not observed when full-length Olig2 protein lacking the basic region was expressed (Novitch et al., 2001). Fusion of the bHLH domain of Olig2 to the transcriptional activation domain of the viral protein VP16 (VP16-Olig2) also appeared to act as a dominant-negative form. When VP16-Olig2 was overexpressed a significant reduction in MNR2/HB9^+^ cells are observed in the ventral neural tube where En1^+^ interneurons are normally produced (Mizuguchi et al., 2001). These observations signify that endogenous Olig2 is pivotal for motor neuron generation and that Olig2 functions as a DNA binding-dependent transcriptional repressor.

#### Inhibitory Interneuron Specification

While the role of Olig2 in motor neuron specification and differentiation is well characterized, less is known of its function in interneuron generation. Early clues suggesting that Olig2 regulates interneuron production is observed by their unique expression patterns in the embryonic brain. In the E13.0 embryonic mouse brain, *Olig1* expression was minimal and particularly restricted in the hypothalamus. *Olig2* expression, on the other hand, was broader and demarcated specific boundaries of brain such as the hypothalamus and the VZ and SVZ of the LGE and MGE (Takebayashi et al., 2000; Ono et al., 2008). Compared to the LGE, a greater number of *Olig2*^+^ cells were observed in the MGE, a region known to give rise to cortical interneurons, from E9.5 to E16.5 (Takebayashi et al., 2000; Miyoshi et al., 2007). Genetic fate mapping analysis of Olig2-expressing precursors also further confirmed the role of Olig2 in generating distinct cohorts of interneurons within the MGE at different time points. At early developmental time points from E9.5 to E10.5, Olig2^+^ precursors were found in the deep cortical layers whereas during later time points at E15.5, these cells were situated primarily in the superficial cortical layers. The distribution of interneuron populations was also found to be mutually exclusive for parvalbumin (PV), somatostatin (SST), and vasoactive intestinal polypeptide (VIP) interneurons, however, a subpopulation of interneurons displayed dual labeling of SST with calretinin (CR) and VIP with CR. Throughout development (E9.5–E15.5), 50% of fate-mapped cells were identified as fast-spiking PV-positive interneurons. At early time points, 30% of fate-mapped cells were classified as SST^+^CR^–^ interneurons, which were completely absent at later time points. Finally, VIP^+^ and CR^+^ interneuron subtypes were not fate-mapped at early timepoints but were pronounced by E15.5. Surprisingly, conditional loss-of-function analysis by combining floxed-*Olig2* allele revealed no significant changes in these cortical interneurons. These data suggest that loss of *Olig2* function in interneuron subtype specification may be compensated by other bHLH genes, such as *Olig1*, or that *Olig* genes have no functional roles in interneuron specification (Miyoshi et al., 2007). Future lineage studies with *Olig2*^+^ progenitors will further elucidate the roles of Olig2 in interneuron production.

While it remains to be determined whether *Olig* genes participates in interneuron specification, one study has demonstrated a role of *Olig1* in regulating interneuron production in the adult mouse brain. Compared to WT mice, *Olig1*-null mice exhibited a ∼35% increase in PV^+^ and CR^+^ interneurons, however, there were no differences in the number of SST^+^ and neuropeptide Y^+^ (NPY) interneurons between WT and *Olig1*-null mice. *Olig1*-*cre* precursors also fate mapped to ∼35% of GABAergic and ∼45% of PV^+^ interneurons (Silbereis et al., 2014). These findings are in line with the fact that SST^+^ and NYP^+^ interneurons are produced prior to the onset of oligodendrocyte specification whereas PV^+^ and CR^+^ cells are derived during late embryogenesis (Miyoshi et al., 2007). *In situ* hybridization of *Olig1* mutants also revealed an increase in *Dlx1* an *Dlx2* expression in the ventral MGE and anterior entopeduncular area (AEP), regions where cortical neurons are produced (Silbereis et al., 2014). *Dlx1* and *Dlx2* are part of the Dlx family of homeobox transcription factors that regulates GABAergic interneuron differentiation, migration, and formation (Sultan et al., 2013). *Dlx1/2* were also found to promote neurogenesis in the MGE and AEP by negatively regulating *Olig2*-dependent OPC formation (Petryniak et al., 2007). It was further demonstrated that *Olig1* is a direct repressor of *Dlx1/2* at the *I12b* intergenic enhancer region. The *I12b* enhancer contains three E-box sites and purified Olig1 protein showed an affinity for E-box 1. Specificity of Olig1 binding to enhancer DNA sequences was determined using supershift assays where binding was inhibited with an antibody against Olig1 but not with control IgG antibody. To confirm that Olig1 acts as a transcriptional repressor, *I12b* enhancer was cloned into the pGL4 luciferase construct and transfected into P19 embryonal carcinoma cells. Dlx2 expression was then transfected to induce *I12b*-dependent luciferase activity. When Olig1 expression construct was transfected into P19 cells, *Dlx2*-induced *I12b* luciferase activity were significantly reduced to 40% of control levels. Furthermore, conditional deletion of *Dlx1/2* from the Olig1 expression domain led to increased production of PV interneurons and GABAergic interneurons in *Olig1*-null mutants *in vivo* and *in vitro*, respectively (Silbereis et al., 2014). Overall, these findings indicate that Olig1 regulates the neural-glial switch by promoting genesis of interneurons by acting as a transcriptional repressor of Dlx1/2.

Two sets of GABAergic neurons are derived from the Olig3 lineage in the ventricular zone of the developing cerebellum: (1) Foxp2^+^ Purkinje cells between E11.5 and E13.5 and (2) Pax2^+^ inhibitory interneurons between E14.5 and P0. Ablation of Olig3 led to a large decline in Foxp2^+^ cells indicating its role in specifying GABAergic Purkinje cells, however, loss in Purkinje cell numbers were not detected until after E13.5. Surprisingly, however, an increase in Pax2^+^ inhibitory interneurons were observed in the absence of Olig3. The number of Ptf1a^+^ cells were also similar in both control and Olig3 mutant mice in the ventricular zone where both proliferative and apoptotic cells did not differ between the two genotypes. The increase in Pax2^+^ inhibitory interneuron was found to be transformed from misspecified Purkinje cells in the absence of Olig3. The development of Foxp2^+^ Purkinje cells and Pax2^+^ inhibitory interneurons were assessed using a knockin mouse that expresses GFP from the Olig3 locus. Compared to WT and heterozygous Olig3^GFP/+^ mice, which expressed Pax2^+^ in the rostral domain of the ventricular zone that lacked Olig3 expression, Olig3^GFP/GFP^ mutant mice displayed excessive Pax2^+^ cells at E13.5. Additionally, in Olig3 mutant mice, 52% of Foxp2^+^ cells coexpressed Pax2 while 90% of Pax2^+^ coexpressed Fox2 at E13.5 which declined by E14.5. The loss of Foxp2^+^/Pax2^+^ cells overlapped with an increase in Foxp2^–^/Pax2^+^ inhibitory interneurons. These findings suggests that Olig3 represses Pax2 during early cerebellar development and that Foxp2^+^/Pax2^+^ cells were misspecified in Olig3 mutant mice and adopted an inhibitory interneuronal fate (Lowenstein et al., 2021).

#### Excitatory Neuron Generation

Although Olig2^+^ cells were shown to primarily differentiate into inhibitory neurons (Miyoshi et al., 2007; Ono et al., 2008), studies have established that Olig2 can also give rise to excitatory glutamatergic neurons (Ono et al., 2008; Kim et al., 2019). The first evidence that Olig2-expressing cells can transform into excitatory neurons was demonstrated in mice using a tamoxifen-inducible Cre/loxP system. In P7 cortical regions following E12.5 tamoxifen treatment, ∼2% of recombinant cells expressed *VGlut1* in the dorsal or ventral pallium. Interestingly, a substantial number of recombinant cells expressed *VGlut2* in both the peri and postnatal stages. In E17.5 caudal hypothalamus, ∼31% of recombinant cells were positive for *VGlut2* following E9.5 and E10.5 tamoxifen treatment. In the adult brain, *VGlut2* expression was also observed in the caudal hypothalamus following E12.5 tamoxifen treatment, however, they do not exhibit the typical pyramidal profile of excitatory neurons (Ono et al., 2008). These glutamatergic cells may have originated from embryonic Olig2^+^ progenitors in the hypothalamus. Indeed, Olig2 expression have been observed in embryonic stages in both mice (Ono et al., 2008) and human (Jakovcevski and Zecevic, 2005).

Recently, Olig2 derived glutamatergic neurons were identified in human Olig2-expressing neural progenitor cells (NPCs). Using Olig2-GFP knockin hPSC reporter lines, dorsal forebrain organoids (DFOs) and ventral forebrain organoids (VFOs) were generated to examine the temporal expression of Olig2 and their cell fate. Similar to earlier *in vivo* findings in mice (Ono et al., 2008), Olig2^+^ cells were abundant in VFOs with only a small subset of Olig2^+^ cells in the DFOs. Using two-photon Ca^2+^ imaging, cells from the DFOs exhibited significantly shorter duration of Ca^2+^ transient and significantly greater number of Ca^2+^ oscillations and peak value, characteristics of “neuron-like” cells. Additionally, GFP^+^ cells in DFOs exhibited greater immunoreactivity to βIII-tubulin (βIIIT), coexpressed the immature neuronal migration protein double cortin (DCX) in the processes, and some were TBR2^+^, a marker for intermediate neuronal progenitors, further confirming neuronal differentiation from Olig2^+^ cells. VGlut1^+^ puncta were also observed along the cell processes suggesting formation of glutamatergic synapses. Indeed, a subset of GFP + βIIIT + cells also expressed glutaminase, an enzyme that catalyzes the production of glutamate from glutamine. These findings provide evidence supporting that human Olig2^+^ NPCs are also able to give rise to excitatory glutamatergic neurons.

Ablation of *Olig3* confirmed its role in the generation of DCN and EGL cells. *Olig3* mutant mice not only exhibited severe cerebellar hypoplasia but also displayed reductions in excitatory neuronal markers Tbr1 and Brn2. Short-term lineage tracing experiments further revealed significant decline in DCN and EGL cells in *Olig3* mutant mice compared to control mice. Additionally, Atoh1^+^ and Brdu^+^ cells in the rhombic lip were also reduced suggesting that Olig3 plays a role in cell proliferation in the rhombic lip and that its function is distinct from that in the ventricular zone (Lowenstein et al., 2021).

### Astrocyte Formation

It is now well established that oligodendrocytes and neurons all arise from Olig^+^ precursor cells. This raise the question on whether astrocytes also emerge from the same cellular lineage. While several studies have attempted to address this question, the findings have been contradicting.

To begin to understand the relationship between astrocytes and *Olig* genes, *in situ* hybridization studies were performed to identify cell types that expressed both *Olig* genes and astrocyte markers. In the postnatal mouse brain, Olig1/2 mRNA did not colocalize with the mature astrocyte marker glial fibrillary acid protein (GFAP). Astrocyte marker expression along with Olig1 and Olig2 expression were also mutually exclusive in the rat optic nerve which is highly abundant in oligodendrocytes and astrocytes. In the optic nerve of P14 rats, Olig1 and the astrocyte marker S100β were not coexpressed (Lu et al., 2000). Similarly, Olig2 was not expressed by astrocytes in the optic nerve of P5 rats (Zhou et al., 2000). These findings imply that astrocytes do not share a common precursor.

Although *Olig* gene expression was not found in astrocytes, studies have proposed that they may participate in astrocyte specification. As mentioned above, *Olig1/2* double mutants embryos lack OPCs. In the spinal cord of these mice, there was no increase in apoptotic cells from E12.0 to E14.0 ruling out the possibility that cell death resulted in the absence of OPCs. To determine whether cellular respecification occurred, the *Olig2* knockin marker hGFP was used as a short-term lineage tracer to compare the fate of Olig2^+^ progenitors in the presence or absence of *Olig1/2* function. In the heterozygous spinal cord of E13.5 mice, GFP^+^ precursors exhibited similar migrating patterns to that of endogenous *Olig2* where GFP labeling was apparent in both somatic and visceral motor neurons. In contrast, the distribution of Olig2-hGFP-expressing cells in homozygous mutants were altered in various aspects. For instance, GFP^+^ precursors displayed minimal migration into the gray matter even though there were similar number of GFP-expressing cells in the homozygous spinal cord compared to the heterozygous spinal cord. Interestingly, a reduction in GFP^+^ cells was observed by E16.5 and E18.5 which was attributed to decrease in cellular proliferation rather than cell death. Furthermore, GFP^+^ cells were shown to move toward the pial surface of the ventral white matter in *Olig1/2*^–/–^ mice by E18.5 where they may possibly transform into astrocytes. Indeed, many GFP^+^ cells in this region were found to coexpress GFAP or S100β, as compared to *Olig1/2*^±^ mice, suggesting conversion of Olig2-hGFP precursors into astrocytes. Double labeling of GFAP or S100β with GFP^+^ cells of the spinal cord was further quantified in *Olig1/2*^–/–^ and *Olig1/2*^±^ mice. In the homozygous spinal cord, over 50% of GFP^+^ cells coexpressed with GFAP and 44–66% of GFP^+^ cells coexpressed with S100β. In stark contrast, none of the GFP^+^ cells coexpressed with GFAP and less than 10% of GFP^+^ cells coexpressed with S100β in the heterozygous spinal cord. Moreover, *Olig1/2*^±^ spinal cord developed oligodendrocytes with colocalization of O4 and GFP which was not observed in *Olig1/2* double mutant spinal cord (Zhou and Anderson, 2002). These findings suggest that lack of *Olig1/2* functions can lead to *Olig2*-expressing cells to promote astrocyte differentiation.

It is likely that astrocytes generated in *Olig1/2* double mutants have gone through sequential transformation. In the normal developing neural tube, astrocytes (which are produced after V2 interneurons) are generated from the p2 domain (Rowitch, 2004; Kessaris et al., 2008). In *Olig1/2* double mutants, the p2 domain expands into the pMN domain where the pMN domain is essentially lost. This results in the generation of V2 interneurons and astrocytes rather than motor neurons and oligodendrocytes (Rowitch, 2004). The invasion of the pMN territory by the p2 domain may also result in de-repression of *Irx3* (Anderson et al., 2002) which is actively repressed by *Olig* genes (Novitch et al., 2001). The upregulation of *Irx3* expression can thus not only inhibit motor neuron differentiation but also promote astrocyte differentiation. Because Olig2-hGFP precursors in *Olig1/2*^–/–^ mice generated astrocytes rather than oligodendrocytes (Zhou and Anderson, 2002), this may suggest that during normal development *Olig2* precursor cells produces astrocytes (Anderson et al., 2002). However, this is unlikely because hGFP lineage tracer was not detected in astrocytes in *Olig1/2* heterozygotes (Zhou and Anderson, 2002). Additionally, fate mapping experiments did not detect any astrocytes in *Olig1*-expressing mice. Using Cre recombinase, *Olig1-cre* mice were generated and daughter cells were investigated at later stages of development in the optic nerve and the brain. In the spinal cord at E10.5 and E12.5, β-galactosidase (β-gal; indicating restoration of β-gal activity in cells expressing *cre*) were coexpressed with *Isl1* and *Hb9* (for motor neurons) and with *PDGFR*α and *Sox10* (for oligodendrocytes). Additionally, colocalization of β-gal and CC1, a marker for mature oligodendrocyte, was observed in both the optic nerve and brain. In contrast, β-gal immunoreactivity did not colocalize with S100β in the optic nerve and brain (Lu et al., 2002). These findings provide evidence that although *Olig*-expressing cells have the potential to differentiate into astrocytes, the actual cellular fate of *Olig*^+^ precursors remain neurons and oligodendrocytes.

While the aforementioned studies indicate the bipotential of *Olig* genes, additional findings have argued that *Olig* genes, specifically *Olig2*, possess tripotential capabilities in the mammalian forebrain. Furthermore, it was suggested that Olig2 may not possess a functional role in interneuron specification at all (Miyoshi et al., 2007). To corroborate these findings, experiments using retroviral-mediated gene transduction to express normal, dominant, and interfering forms of *Olig2* in SVZ cells were employed. First, transduction of SVC cells *in vivo* with GFP using a replication-incompetent retrovirus encoding GFP (control virus encoding *eGFP* alone; *X-ires-eGFP*) found that Olig2 was expressed exclusively by glial cells, most which had migrated into the white matter, striatum, and cortex and displayed morphologies reminiscent of oligodendrocytes and immature astrocytes. Infected cells that migrated along the RMS and situated in the olfactory bulb did not express Olig2 and were closely aligned with Olig2^–^ migratory neuroblasts. Second, transduction of SVZ cells with bistronic retrovirus encoding *Olig2* and *eGFP* (*Olig2-ires-eGFP*) did not label any cells in the distal RMS or olfactory bulb suggesting that Olig2 expression in SVZ cells prevented the migration and differentiation of olfactory interneurons. Additionally, cells infected with *Olig2-ires-GFP* appeared to specify SVZ cells toward glial lineage where over 70% of the infected cells displayed morphology of oligodendrocytes and expressed NG2 and 20% of infected cells had characteristics of astrocytes and expressed GFAP. The remaining 10% of infected cells exhibited glial morphologies but did not express markers for oligodendrocytes, astrocytes, or neurons. Finally, loss of Olig2 function experiments were conducted to confirm that Olig2 expression influences glial cell specification. SVZ cells transduced with an Olig2 construct containing the bHLH domain alone (*Olig2bHLH-ires-eGFP*) were found to migrate along the RMS, olfactory bulb, white matter, and cortex but with less than 5% of infected cells expressing GFAP or CC1. Additionally, severe disruption in glial differentiation was observed in over 90% of infected cells that colonized the white matter and cortex. Interestingly, ∼25% of these cells ectopically expressed the neuronal marker NeuN (Marshall et al., 2005). These findings indicate that Olig2 function in SVZ cells is required for astrocyte and oligodendrocyte formation but not for interneuron development.

Differentiation of specific subtypes of astrocyte has also been observed in Olig2-expressing human NPCs. Spontaneous generation of S100β^+^ astrocytes from Olig2^+^ NPCs has also been observed in brain organoids derived from Olig2-GFP hPSC reported lines (Kim et al., 2019; Xu et al., 2019). Induction of specific subtypes of astrocytes from Olig2^+^ NPCs or glial progenitor cells (GPCs) can also be achieved by activating specific signaling pathways. For example, human embryonic GPCs isolated from 9.5 week old spinal cords treated with either bone morphogenetic protein (BMP) or ciliary neurotrophic factor (CNTF) promoted two distinct groups of astrocytes. Interestingly, astrocytes derived from GPCs cultured in BMP resulted in repression of Olig2 whereas those cultured in CNTP expressed high levels of Olig2 (Davies et al., 2011). Similarly, Olig2-GFP hESCs cultured in BMP or CNTF also led to the development of two separate subtypes of astrocytes that expressed GFAP and S100β and with Olig2 transcript largely absent (Jiang et al., 2013). Interestingly, astrocytes cultured with BMP were also found to have superior neuroprotective effects compared to those that were treated with CNTP. For example, in a rat spinal cord injury model, transplantation of GPCs cultured in BMP led to improved functional outcome as well as significantly increased numbers of neurons around the injury site (Davies et al., 2011). In a rat global cerebral ischaemia model, transplantation of astrocytes derived from hESC-derived Olig2-expressing NPCs also resulted in increased numbers of neurons and improved behavioral recovery (Jiang et al., 2013). These findings suggest that specific populations of human astrocytes with distinct biological and functional properties can be generated from Olig2^+^ NPCs contributing to overall astrocyte heterogeneity. More importantly, transplantation of these cells may be a potential cell replacement therapy for CNS injuries.

## The Olig Family in Developmental Disorders

Because of its influences on neurodevelopment, the Olig family of transcription factor are key targets in CNS developmental disorders. Here, we describe well recognized developmental diseases with possible connections to the Olig family: 1) DS and 2) ASD.

### Down Syndrome

Down syndrome, caused by triplication of human chromosome 21 (Hsa21), is the most common genetic cause of intellectual disability (Epstein, 1989; Patterson, 2009). In addition to cognitive and learning deficits, individuals with DS exhibit distinctive facial abnormalities and many are born with congenital heart disease and have increased risk of leukemia, early-onset Alzheimer’s disease (AD), and immune defects (Epstein, 1989; Korenberg et al., 1994; Wiseman et al., 2009). Although the mechanisms underlying impaired cognition remains unclear, multiple studies have found altered early brain development associated with DS (Haydar and Reeves, 2012). For example, trisomy 21 resulted in delayed and disorganized cortical development (Golden and Hyman, 1994), gross brain malformations and decreased brain weight (Schmidt-Sidor et al., 1990), dendritic atrophy (Marin-Padilla, 1976; Becker et al., 1986; Weitzdoerfer et al., 2001), and loss of neurons and synapses (Kurt et al., 2004; Vacca et al., 2019). Both *Olig1* and *Olig2* are located on Hsa21 and triplication of Hsa21 induces *Olig* genes overexpression that may contribute to DS phenotypes (Xu et al., 2019).

#### Consequences of *Olig* Overexpression in DS

The genetic predisposition for DS remains to be determined as more than 500 genes present on Hsa21 are thought to be dysregulated (Moyer et al., 2020). However, recent findings have discovered a link between the *Olig* genes that may have a direct impact on DS (Chakrabarti et al., 2010; Liu et al., 2015; Xu et al., 2019). Overexpression or misexpression of *Olig1*/*2* is a common feature in experimental models of DS (Chakrabarti et al., 2010; Lu et al., 2012; Xu et al., 2019). Indeed, a 1.7- and 1.5-fold increase in *Olig1* and *Olig2* expression, respectively, in the MGE of the ventral telencephalon was observed in the widely used Ts65Dn mouse model of DS at E14.5. Moreover, Ts65Dn mice exhibited significantly more *Olig2*^+^ cells compared to euploid littermates at E13.5 and E14.5 (Chakrabarti et al., 2010). Interestingly, removal of one allele of each gene in the Ts65Dn background was shown to return neurogenesis to euploid levels. For instance, the percentage of BrdU^+^ neurons in the MGE was significantly reduced in Ts65Dn *Olig1/2*^±^ mice compared to Ts65Dn mice and the percentage of MGE BrdU^+^ neurons of Ts65Dn *Olig1/2*^±^ was comparable to those of euploid mice. The fraction of cells exiting cell cycle was significantly increased in the VZ and SVZ of the MGE in Ts65Dn mice compared to euploid controls, however these levels returned to euploid levels in Ts65Dn *Olig1/2*^±^ mice. Furthermore, *Olig2*^+^ cells in the MGE VZ and SVZ of Ts65Dn *Olig1/2*^±^ mice was comparable to those of euploid mice. These findings indicate that normalization of *Olig1* and *Olig2* gene copy number was sufficient to reduce neurogenesis in the MGE and rescue Ts65Dn phenotype (Chakrabarti et al., 2010).

Olig1 and Olig2 overexpression was also noted in DS human NPCs. Compared to age-matched controls, a significant upregulation of Olig2 and PDGFRα expression and Olig1, Olig2, and PDGFRα expression were observed in 14 week gestational age (GA) and 18 week GA DS frontal cortices, respectively. The upregulation of these OPC markers are thought to contribute to impaired cellular proliferation. However, in contrast to the mice studies mentioned above, a 3 to 4-fold decrease in Ki67 and the M-phase marker phosphor-histone H3 (PH3) immunoreactivity was observed along the VZ in 14–18 week GA DS frontal cortex tissue compared to age-matched controls. DS neurospheres derived from the VZ of 18 week GA DS frontal cortices displayed significant reductions in Ki67^+^ and BrdU^+^ cells compared to controls (Lu et al., 2012). This discrepancy may be attributed to different species and regions of the brain under investigation. Additionally, it is thought that decreases in voltage-gated potassium currents contribute to the impaired cellular proliferation observed. Several studies have suggested that voltage-gated potassium channels play a role in cell growth and proliferation (Pardo, 2004). DS HNPs exhibited reductions in voltage-gated potassium currents compared to controls that were correlated to reduced expression of the voltage-gated outward potassium channel KCNA3. Moreover, overexpression of Olig2 in DS HNPs infected with lentivirus carrying Olig2 construct significantly downregulated expression of KCNA3 and proliferating cell nuclear antigen (PCNA). Similar decrease in KCNA3 and PCNA expression was also observed in the cortex of E14.5 transgenic mice overexpressing Olig2. On the other hand, lentiviral infection with human *Olig2* shRNA upregulated the expression of KCNA3 and PCNA and increased expression of Olig1, possibly as a compensatory mechanism. Finally, blocking KCNA3 led to significant reductions in BrdU^+^ and Ki67^+^ in cultured HNPs, DS HNPs, and mouse neural progenitors (Lu et al., 2012). Altogether, these findings suggest that triplication of Olig2 can result in dysregulation of KCNA3 leading to cellular growth and proliferation deficits.

Recently, a correlation between neurogenesis and Olig2 overexpression was proposed. Using the *in vivo* Cre/loxP system, a transgenic mouse line with Olig2 overexpression in nestin-expressing NPCs was generated. These inducible transgenic mice (iTg-Nes) have significantly reduced brain thickness, disrupted cortical lamination, massive neuronal cell death, and impaired locomotion. Not surprisingly, iTg-Nes mice also display marked Olig2 expression in the cortex at E18.5 compared to iTg controls. Using quantitative RT-PCR (qRT-PCR), an ∼3 to 6-fold increase in Olig2 expression was revealed as compared to age-matched controls. At postnatal day (P) 7, iTg-Nes mice exhibit robust increase in PDGFRα^+^ OPCs in the developing cortex and midbrain as well as a significant increase in Sox10^+^ oligodendrocyte lineage cells in the midbrain, suggesting that overexpression of Olig2 promotes differentiation of NPCs into oligodendrocyte lineage cells. Similarly, iTg-Nes mice exhibit defects in cell growth and proliferation. Specifically, significant reductions in cortical Ki67^+^ and BrdU^+^ cells were detected in the iTg-Nes mice compared to controls. Furthermore, cell cycle progression of cortical progenitors was also impaired where a significant decrease in the percentage of cells remained in the S-phase while a significant increase in the percentage of cells that exited the cell cycle was observed. ChIP-seq revealed Olig2 occupied promoter or enhancer regions of *Nfatc4*, *Pax6*, *Dyrk1a*, and *Dscr1/Rcan1*, genes that regulate neurogenesis and are associated with DS phenotypes. Gene expression analysis confirmed impaired neurogenesis where a significant downregulation of proneural and neuronal differentiation genes were detected at E14.5 and *in situ* hybridization analysis further identified severe reductions of pro-neurogenic genes in the cortex (Liu et al., 2015). Thus, overexpression of Olig2 are associated with characteristics of DS.

Abnormal expression of Olig2 in human induced pluripotent stem cells (hiPSCs) derived from DS patients was also reported. Here, VFOs were generated from control and DS hiPSCs (Xu et al., 2019). Consistent with previous findings, a significant percentage of Olig2^+^ cells were observed in DS hiPSCs organoids (∼70%) compared to control organoids (∼40%) at week 5 in culture. Moreover, DS hiPSC-derived organoids exhibited a significantly higher expression of *Olig2* mRNA and protein. *Olig1* transcripts were also significantly increased, interestingly however, few Olig1^+^ cells were observed and Olig1 protein expression levels in DS hiPSCs organoids were similar to that of control organoids. These findings establish a potential role of Olig2 in early-stage neuronal differentiation in human cells (Xu et al., 2019).

#### Increased Inhibition by Olig2 Overexpression in DS

The overexpression of Olig2 (Chakrabarti et al., 2010; Lu et al., 2012; Xu et al., 2019) may contribute to the hypothesis that increased synaptic inhibition underlies memory and cognitive impairments in DS (Haydar and Reeves, 2012). In fact, several lines of evidence have demonstrated enhanced inhibition in the Ts65Dn mice. For example, Ts65Dn mice have overproduction of interneurons in the neocortex and hippocampus (Chakrabarti et al., 2010), overexpress G-protein coupled inwardly rectifying potassium (GIRK) channels (Best et al., 2012), exhibit significantly increased inhibitory postsynaptic currents (IPSCs) in the hippocampus (Kleschevnikov et al., 2004; Chakrabarti et al., 2010), and impaired long-term potentiation (LTP) (Kleschevnikov et al., 2004; Costa and Grybko, 2005). In P15 Ts65Dn mice, a remarkable increase in SST^+^ and PV^+^ interneurons in the neocortex and hippocampus was observed compared to euploid controls. Interestingly, there were no significant differences in CR^+^ and calbindin (CB)^+^ interneurons in the neocortex between Ts65Dn and euploid mice. The overproduction of interneurons thus resulted in increased frequency of IPSCs. Reduction in gene dosage was shown to rescue the inhibitory neuron phenotype. In embryonic Ts65Dn *Olig1/2*^±^ telencephalon a significant reduction in SST-positive cells comparable to those of euploid controls were observed. Postnatal Ts65Dn *Olig1/2*^±^ mice also displayed near normal numbers of STT^+^ and PV^+^ interneurons in the neocortex. Functionally, the frequency of IPSCs in Ts65Dn *Oig1/2*^±^ mice were similar to those of euploid controls (Chakrabarti et al., 2010).

Increased inhibitory neurons were also seen in the DS hiPSCs VFOs. In contrast to the previous study, 5–6 week old organoids had highest expression of CR^+^ interneurons although strong expression of CB, PV, SST, and NPY interneurons were detected as well. By 8 weeks, ∼60% of cells were GABAergic cells in DS organoids compared to control organoids which only generated ∼35% of inhibitory neurons. DS hiPSCs organoids also exhibited significant more CR^+^ and SST^+^ interneurons compared to control organoids, however no significant differences in the percentage of NPY^+^, CB^+^, and PV^+^ inhibitory cells were observed. These *in vitro* findings were then compared to human DS and control human brain tissue of <1 year old. In DS brains, CR and GAD65/67 protein levels were significantly higher and an increase trend in SST protein levels were observed compared to control brains. To further validate the enhanced expression of GABAergic cells *in vivo*, DS neuronal chimeric mice were generated by dissociating DS organoids into single cells and engrafting the cells into mice brains. The donor-derived cells were then tracked by staining human nuclei (hN). At 6 months post transplantation, the DS chimeric mice exhibited greater percentage of Olig2^+^/hN^+^ and PDGFRα^+^/hN^+^ OPCs compared to control mice. hN^+^ cells also efficiently differentiated into GABAergic interneurons which also coexpressed vesicular GABA transporter (VGAT). DS chimeric mice also had significantly higher percentages of CR^+^/hN^+^ and SST^+^/hN^+^ neurons compared to control mice. No significant differences in CB^+^/hN^+^, PV^+^/hN^+^, and NPY^+^/hN^+^ inhibitory neurons were observed between DS chimeric and control mice. Remarkably, inhibition of Olig2 significantly reduced the percentages of interneurons. RNAi knockdown on DS hiPSCs expressing Olig2 short hairpin RNA (shRNA) were cultured and organoids were also used to generate chimeric mice. In DS organoids with Olig2 shRNA a significant reduction in GABAergic cells were noted with a greater decrease in the percentages of CR^+^ and SST^+^ cells. Blocking of Olig2 in chimeric mice led to a significant increase in neurons as well as a return of GABA immunoreactivity to those of control levels (Xu et al., 2019).

The mechanisms behind Olig2 overexpression and enhanced interneuron production in DS were also explored. RNA-seq detected abnormal gene expression in 5 week old DS hiPSCs where Hsa21 had the greatest percentage of differentially expressed genes (DEG) in which all were upregulated. Inhibiting Olig2 by introduction of Olig2 short hairpin RNA (shRNA) in DS hiPSCs reversed the DEGs compared to controls. Gene ontology (GO) further revealed that downregulated genes in DS organoids were enriched in pathways related to neuronal development which were rescued by blocking Olig2. Not surprisingly, RNA-seq detected a striking increase in transcription factors that regulate interneuron which was reversed after Olig2 knockdown.

#### Neuroinflammation-Mediated Induction of Olig2 Overexpression

The induction of *Olig* gene overexpression is largely due to its location on human chromosome 21 and its triplication in DS. As stated above, various studies using mouse models of DS have attributed *Olig* overexpression to increased inhibitory neurons, excitation/inhibition imbalance, and behavioral deficits (Chakrabarti et al., 2010; Liu et al., 2015; Xu et al., 2019). Similarly, *Olig* overexpression was also observed in DS hiPSCs (Xu et al., 2019). However, it is plausible that *Olig* overexpression may be further exacerbated by neuroinflammation in the brain, a common pathological feature observed in DS (Wilcock, 2012; Flores-Aguilar et al., 2020). The inflammatory process is primarily facilitated by activation of glial cells in response to various cues (Yang and Zhou, 2019). Reactive astrocytes generally occur following a CNS insult and are likely contributors of Olig2 overexpression in DS. For instance, significant upregulation of Olig2-expressing cells have been observed in preclinical models of acute and chronic brain injuries (Buffo et al., 2005; Chen et al., 2008; Amankulor et al., 2009) and experimental autoimmune encephalomyelitis (EAE) (Cassiani-Ingoni et al., 2006). The generation of reactive astrocytes in response to an immune response may involve the translocation of Olig2 into the cytoplasm. Nuclear export of Olig2 to the cytoplasm of GFAP^+^ astrocytes have been detected *in vivo* (Setoguchi and Kondo, 2004) and as previously reported, loss of *Olig2* gene resulted in generation of astrocytes (Rowitch, 2004). In a mouse model of EAE, a subset of cytoplasmic Olig2^+^ cells were found to coexpress both Nkx2.2 and GFAP suggesting that immune-mediated injury promoted the differentiation of reactive astrocytes from progenitor cells (Cassiani-Ingoni et al., 2006). Interestingly, it was found that reactive astrocytes also produce Shh (Becher et al., 2008; Amankulor et al., 2009), which regulates *Olig1* and *Olig2* expression during development (Lu et al., 2000; Tekki-Kessaris et al., 2001) as well as cell proliferation (Becher et al., 2008). After cortical freeze injury, Shh signaling was also found to be maximally expressed and colocalized with GFAP^+^ reactive astrocytes. Inhibition of Shh with cyclopamine led to reduction of both proliferating Olig2-expressing progenitors as well as the total number of Olig2^+^ cells in the injured brain (Amankulor et al., 2009). Therefore, neuroinflammation in DS brains may drive a positive feedback loop in which inflammatory mediators increase proliferation of reactive astrocytes, which express Shh that could induce olig2 expression in progenitor cells as well as glial cells.

### Downregulation of Olig Genes in DS

Although the contribution by Olig2 overexpression to increased inhibition in DS is certainly convincing, recent transcriptomic and proteomic profiling revealed contrasting findings. Using induced pluripotent stem cell (iPSC)-derived neural cell models, molecular perturbations during neurogenesis in DS were detected. Specifically, trisomic NPCs exhibited a 6–7-fold downregulation in Olig1 and Olig2 when compared to euploid NPCs (Sobol et al., 2019). The downregulation of *Olig1* and *Olig2* can thus affect oligodendrocyte function. In fact, six DEGs involved in myelination process were identified (Sobol et al., 2019) which may explain the impaired myelination observed in DS patients (Ábrahám et al., 2012) which can manifest into learning and cognitive disabilities. In fact, transcriptome analysis of postmortem DS and Ts65Dn brains revealed dysregulation of genes associated with oligodendrocyte and myelin formation (Olmos-Serrano et al., 2016). The differences in *Olig* regulation may be explained in part by the stages of differentiation. For example, defective genes correlated with oligodendrocyte function in DS was observed during late fetal development (Olmos-Serrano et al., 2016), a time in which oligodendrocyte and myelin genes are upregulated (Kang et al., 2011). Certainly, gene expression dysregulation have been shown to occur in a spatiotemporal manner in DS (Sobol et al., 2019). Thus, the upregulation of Olig2 mentioned previously may have been observed during neurogenesis (which occurs before oligodendrocyte formation) resulting in an increase in inhibitory neurons.

#### Epigenetic Regulations of Olig2 in DS

While the findings above implicated a role of Olig2 in DS, it remains to be determined how trisomy 21 disrupts neurodevelopment. Epigenetic mechanisms, which regulate gene expression without altering the DNA sequence (Jakovcevski and Akbarian, 2012), is a plausible explanation. Complex interactions amongst genes both on and outside Hsa21 can lead to transcriptional network perturbations. Recently, trans-acting epigenetic effects of chromosomal aneuploidy on DNA methylation of CpG sites have gained interest. These modifications can be passed on to daughter cells during somatic cell division and certain gene expression changes can therefore potentially be transmitted to the next generation (El Hajj et al., 2016; Do et al., 2017; Lim et al., 2019). Although CpG sites were found to be hypermethylated in fetal DS brain tissue (El Hajj et al., 2016; Lu J. et al., 2016; Lim et al., 2019; Laan et al., 2020), changes in methylation states on chromosome 21 appeared to balance between hypo- and hyper-methylation (El Hajj et al., 2016; Lu J. et al., 2016; Do et al., 2017; Lim et al., 2019; Laan et al., 2020). Interestingly, enrichment of hypomethylated sites on chromosome 21 demonstrated strong enrichment of binding sites recognized by Olig2 (Do et al., 2017). Clues as to why or how Olig2 occupancy on hypomethylated CpG sites affects gene expression in DS may be gathered by studies in cancer. While relatively less explored, global genome hypomethylation have been widely reported in various cancers (Hoffmann and Schulz, 2005; Ehrlich, 2009; Mahmood and Rabbani, 2019). Furthermore, overexpression of Olig2 have been detected in various malignant cell lines including gliomas, non-small cell lung carcinoma, melanoma, breast cancer, and leukemia (Marie et al., 2001; Ohnishi et al., 2003; Lin et al., 2005; Ligon et al., 2007; Mehta et al., 2011; Lu F. et al., 2016). In brain cancer, critical gene networks involving Olig2 have been shown to be related to epigenetic regulation such as methyl CpG-binding domain protein 3 (MBD3), a transcriptional repressor and gene silencer for methylated CpG dinucleotide containing sites, and histone deacetylase 7 (*HDAC7*), an epigenetic repressor responsible for transcriptional regulation, cell cycle progression, and development (Tsigelny et al., 2016). Genetic modulatory networks involving Olig2 in DS necessitate further exploration.

### Autism Spectrum Disorder

Autism spectrum disorder is a group of neurodevelopment disorder characterized by impaired social and communication interactions along with restricted repetitive sensory motor behavior. Diagnosed in more than 1% of children, ASD is highly heterogeneous as clinical presentation can include individuals with severe intellectual disability or individuals that are highly functioning with above average intelligence (Grove et al., 2019; Lord et al., 2020). Although various brain structures have been proposed to play a role in ASD symptoms, the etiology and pathogenesis remains unclear. Recently, studies have described an association of Olig2-expressing cells in preclinical models of ASD which may aid in deciphering the underlying mechanisms of this disorder.

#### Oligodendrocytes in ASD

Changes in oligodendrocytes and its functions may be one factor underlying the social and cognitive deficits observed in ASD. Indeed, abnormal white matter integrity has been linked to deficits in social cognition in ASD and alterations in myelin production can result in neural circuit dysfunction (Herbert et al., 2003; Barnea-Goraly et al., 2004). Using a well-characterized mouse model of ASD (induced by prenatal injections of valproic acid; VPA), changes in myelin and oligodendrocyte lineage cells were investigated in P90 mice. Compared to saline treated mice, VPA treated mice exhibited a significant decrease in myelin content that was correlated to a significant decrease in Olig2^+^ cells in the medial prefrontal cortex (mPFC) and the piriform cortex (Pir). Not surprisingly, a concomitant decrease in the mature oligodendrocyte marker CC1 was significantly reduced in the same brain regions in mice treated with VPA (Graciarena et al., 2019). Similar findings were also observed in a rat model of ASD. In P13 VPA treated rats, an increase in Olig2 mRNA was detected in the prefrontal cortex (pFC) and hippocampus compared to saline treated rats. At P35, Olig2 mRNA expression remained high in the pFC in the VPA exposed rats, however, a significant decrease in Olig2 mRNA was observed in the hippocampus. Furthermore, Olig2 protein levels were significantly higher in the pFC at this stage although a significant decrease in Olig2 protein was detected in the cerebellum. Interestingly, by P90, Olig2 mRNA levels were similar in the pFC and cerebellum but was significantly reduced in the hippocampus in VPA treated rats compared to controls. Additionally, Olig2 protein levels were significantly higher in the hippocampus in rats treated with VPA at P90 although Olig2 protein levels were reduced to control levels in the pFC and cerebellum (Bronzuoli et al., 2018). Together, these findings indicate that prenatal exposure to VPA modifies region-specific oligodendrocytes at the transcriptional and translational levels and that loss of oligodendrocytes with concurrent hypomyelination can attribute to ASD-like symptoms.

#### PTEN Mutation and Aberrant Myelination in ASD

Germline mutation in the tumor suppressor gene *phosphatase and tensin homolog deleted on chromosome ten* (*PTEN*) can be one explanation underlying white matter abnormalities in ASD. *PTEN* regulates cell proliferation and survival and is frequently linked to many cancers (Bonneau and Longy, 2000). Recently, studies have found that PTEN mutation can contribute functional changes in myelination in a *Pten*^m3m4^ mouse model, a constitutive knockin model that restricts Pten in the cytoplasm resulting in ASD-like phenotypes. Compared to WT mice (*Pten*^wt/wt^), *Pten* mutant mice exhibited a significant increase in Olig2^+^ OPCs in the cerebral cortex however levels of mature oligodendrocytes were comparable between genotypes. Significant increases in myelin markers were also observed in *Pten*^m3m4^ mice however myelin deposition was improperly deposited and failed to ensheath axons. This can be attributed to the dysfunctional myelinating oligodendrocytes which exhibited abnormal morphology including condensed cell body or fragmented processes (Lee et al., 2019). Compared to controls, Olig2-cre:Pten^fl/fl^ mice, which exhibits Pten loss in Olig2^+^ cells, displayed gross enlargement of the corpus callosum which was attributed to excess myelin wrapping ultimately leading to leukodystrophy (Maire et al., 2014). These studies demonstrate that cellular mislocalization of Pten and dysregulation in Pten signaling can have dramatic effects on oligodendrocyte function. The relationship between Olig2 and Pten, however, remains to be elucidated.

## Olig-Directed Treatments for Neurodevelopment Disorders

Currently, there are no known Olig-directed therapies for treating neurodevelopmental disorders such as DS and ASD. Potential treatment strategies to rescue DS and ASD phenotypes would require strict regulation of Olig1 and/or Olig2 in a temporally and spatially distinct manner. Exploitation of the Olig bHLH transcription factors to affect its downstream targets may also be therapeutically beneficial. Studies have found that both Olig1 and Olig2 are involved in the transforming growth factor beta (TGFβ) signaling pathway (Ikushima et al., 2008; Ikushima and Miyazono, 2012; Motizuki et al., 2013; Singh et al., 2016). TGFβ signaling regulates a host of cellular processes including cell growth, survival, fate specification during embryogenesis and adulthood (Massagué and Chen, 2000; Miyazono, 2000). In brief, TGFβ signaling transduction involves binding of TGFβ ligands to its receptors which initiates phosphorylation and activation of Smad proteins allowing it to translocate to the nucleus for transcription of target genes (Massagué and Wotton, 2000).

It was discovered that Olig1 is a Smad cofactor where it physically interacts with Smad2/3 in response to TGFβ stimulation (Ikushima et al., 2008). The cooperation of Olig1 with Smad2/3 was found to be regulated by a peptidyl-prolyl *cis/trans*-isomerase, Pin1. Compared to control, NMuMG cells transfected with Pin1 SiRNA led to decreased interaction of Smad2/3 with Olig1 (Motizuki et al., 2013). Transcription of target genes via the Olig1-Smad2/3 complex was inhibited by the human homolog of maternal Id-like molecule (HMM) (Ikushima et al., 2008), a dominant negative helix-loop-helix protein that is implicated in gene expression regulation, cell cycle progression, and cell migration and motility (Terai et al., 2000; Motizuki et al., 2013, 2015). Indeed, Olig1 was found to be involved in TGFβ-induced cell mobility. In a chamber migration assay, NMuMG cells transfected with Olig1 siRNA selectively impaired cell motility (Motizuki et al., 2013). Additionally, NMuMG cells transfected with Pin1 siRNA attenuated expression of plasminogen activator inhibitor-type 1 (*PAI-1*) (Motizuki et al., 2013), a protease inhibitory that plays a role in cell signaling and cell migration (Czekay et al., 2011). Interestingly, Olig1-Smad2/3 complex was found to regulate transcription of PAI-1 where its expression is inhibited by HMM (Ikushima et al., 2008).

Prospective Olig-directed treatments for DS or ASD can also be derived from lessons in glioblastoma (GBM), the most aggressive and lethal brain tumor in adults (Holland, 2000). The highly infiltrative nature of GBM is due to upregulated genes involved in cell migration (Loftus et al., 2012; Brennan et al., 2013; Dhruv et al., 2013). Indeed, Olig2 has been shown to promote cell migration/invasiveness in not only glioma stem cells (GSCs) but also in normal OPCs (Hornig et al., 2013; Nevo et al., 2014). Interestingly, findings have found that N-terminal phosphorylation of Olig2 at the triple-serine motif (S10, S13, and S14) can regulate cell migration through the TGFβ2 pathway (Meijer et al., 2014; Singh et al., 2016). Specifically, cells expressing unphosphorylated Olig2 or low levels of phosphorylated Olig2 (pOlig2^low^) were both highly invasive with increased levels of TGFβ2. Additionally murine glioma-like stem cells (mGSCs) expressing triple phosphonull (TPN) and human patient-derived GBM GSCs (hGSCs) expressing pOlig2^low^ were not only substantially infiltrative but also had high expression of *ZEB1* (Singh et al., 2016), an epithelial-like mesenchymal transition (EMT) inducing factor that promotes GBM cell invasion, regulates cell migration, and is a direct genetic target of Olig2 (Siebzehnrubl et al., 2013; Singh et al., 2016; Rosmaninho et al., 2018). Interestingly, phosphorylated Smad2 (pSmad2) were found to be inversely correlated with high levels of pOlig2. For instance, hGSCs with pOlig2^low^ had increased levels of pSmad2 whereas hGSCs with pOlig2^high^ had low expression of pSmad2. It is suggested that unphosphorylated Olig2 initiates expression of TGFβ2 which binds to its respective TGF receptors to phosphorylate Smad2 resulting in its nuclear translocation to increase cell migration. Altogether, these findings suggest that posttranslational modifications of Olig proteins can govern disease progression through the TGFβ pathway.

**FIGURE 3:**
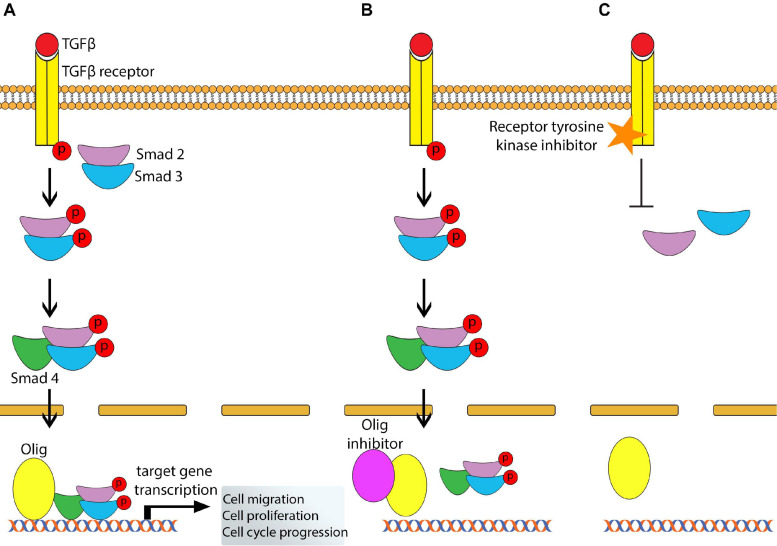
Proposed Olig-directed drug targets. **(A)** Summary of TGFβ signaling. Binding of TGFβ to its TGFβ receptor leads to recruitment and subsequent phosphorylation of Smad2/3 complex. Phosphorylated Smad2/3 recruits Smad4 which translocate to the nucleus and binds with Olig protein to allow gene transcription for specific biological processes (e.g., cellular migration). **(B)** An Olig inhibitor is bound to the Olig protein to prevent its binding to E-box motifs, thus blocking gene transcription. **(C)** A receptor tyrosine kinase inhibitor blocks phosphorylation of the TGFβ receptor preventing TGFβ signaling.

Overall, there may be multiple Olig-targeted treatment strategies for neurodevelopmental disorders (Figure 3). Based on the above findings, one method may be selectively inhibiting Olig1-Smad2/3 or Olig2-Smad/2 to attenuate TGFβ-mediated cell migration. For instance, the increase in interneurons observed in DS may be contributed by not only Olig2 overexpression but also the overall increase in cell migration. Additionally, blocking these bHLH transcriptions factors to form heterodimeric complexes can also prevent transcription of specific target genes that may be associated with the intellectual disabilities observed in both DS and ASD, although these genes remain to be determined (Flint, 2001). Interrogation of the cellular-fate switch in Olig-expressing cells at a definite time may also be another approach. Up or downregulation of Olig1 and/or Olig2 in a temporal manner may allow conversion of cellular lineage that may be therapeutically beneficial. If Olig transcription factors do in fact have tripotential capabilities, pharmacological transformation of the excess interneurons into neurons and/or astrocytes may aid in correcting the excitatory/inhibitory imbalance in DS ultimately modifying cognitive deficits. Additionally, activating Olig1 in ASD may assist with hypomyelination. Genetic targets and signaling pathways regulated by all three Olig proteins requires further investigation for the development of novel compounds.

## Conclusion

The Olig proteins are family members of bHLH transcription factors that dynamically control neurodevelopment to promote cellular heterogeneity. It is well recognized that Olig1 and Olig2 have divergent, non-overlapping roles in cellular patterning and specification, however mechanisms underlying dysfunctional Olig1 and Olig2 leading to aberrant cell identities and functions remain to be determined. In this review, we highlighted the biological roles of the Olig family, particularly Olig1 and Olig2, and how they are implicated in CNS developmental disorders DS and ASD.

The use of genetic techniques has also greatly improved our understanding of the cellular phenotypes that is regulated by Olig1 and Olig2. It is now appreciated that Olig1 and Olig2, along with their combinatorial interactions with other co-factors, promote the specification and differentiation of neurons and glial cells in a region-specific manner. Despite extensive research, many questions remain unanswered. For example, how does Olig2 regulate oligodendrocyte development in the brain? Additionally, Olig3 is severely understudied. For instance, what are the functional roles of Olig3 during postnatal neurodevelopment, how is Olig3 regulated, and does Olig3 act as a transcriptional repressor or activator?

Despite their crucial role in development, little is known on how the Olig members influence neurodevelopmental disorders. It was only recently discovered that Olig2 was implicated in DS and ASD, however, how it directly affects DS and ASD phenotypes is still unclear. Additionally, the contribution of Olig1 and Olig3 to the hallmark symptoms of DS and ASD warrants investigation. The relationship of Olig proteins in other neurodevelopmental disorders such as Fragile X Syndrome (FXS) and attention-deficit/hyperactivity disorder (ADHD) also requires attention. Additionally, the functional role of the Olig family in neurological diseases affecting other Olig lineage cell types such as motor neurons seen in amyotrophic lateral sclerosis (ALS) is of great interest. Finally, understanding how Olig genes and proteins influence inflammatory neurological disorders such as MS can further shed light onto their mechanism of actions.

Because of their relevance in public health, developmental therapeutic opportunities that target the Olig proteins are essential. For instance, small molecule agonists or antagonists of the Olig members may potentially rescue cognitive impairments, however, this is challenging as transcription factors are not ideal drug targets due to their interactions with DNA and other co-factors. Complete identification of genetic targets and signaling pathways affected by changes in Olig expression may not only further our understanding of the Olig family but is another strategy for drug development. For instance, a more attractive pharmacological approach can be aimed at their distinct genetic target or posttranslational modification enzymes. Thus, future research on Olig functions is crucial for the generation of novel therapies that may regulate their expression and activity not only in CNS developmental disorders but also in AD, brain tumors, and demyelinating diseases.

## Author Contributions

SK conceived this manuscript. JS performed a complete literature review and drafted the review manuscript and figures. AW, PJ, and SK provided critical feedback on its content. All authors contributed to the article and approved the submitted version.

## Conflict of Interest

The authors declare that the research was conducted in the absence of any commercial or financial relationships that could be construed as a potential conflict of interest.
